# Synthesis, Spectroscopic, and Anticancerous Properties of Mixed Ligand Palladium(II) and Silver(I) Complexes with 4,6-Diamino-5-hydroxy-2-mercaptopyrimidine and 2,2′-Bipyridyl

**DOI:** 10.1155/2008/723634

**Published:** 2008-04-08

**Authors:** Sahar I. Mostafa, Farid A. Badria

**Affiliations:** ^1^Chemistry Department, Faculty of Science, Mansoura University, 35516 Mansoura, Egypt; ^2^Liver Research Laboratory, Faculty of Pharmacy, Mansoura University, 35516 Mansoura, Egypt

## Abstract

Synthesis of two new water-soluble mixed ligand [Pd(bpy)(dahmp)]Cl and [Ag(bpy)(Hdahmp)]NO_3_ complexes (dahmp and Hdahmp are the deprotonated monoanion and the protonated neutral 4,6-diamino-5-hydroxy-2-mercaptopyrimidine, resp.) is reported. The composition of the reported complexes was discussed on the bases of IR, ^1^H NMR, and mass spectra, as well as conductivity and thermal measurements. The reported complexes display a significant anticancer activity against *Ehrlich ascites* tumor cells (EACs). The higher activity of these complexes with their higher conductivity values corresponds to their complete ionization in aqueous solution.

## 1. INTRODUCTION

Currently, cisplatin is being used as an anticancer agent in several human cancers, particularly, testicular and ovarian cancers [1, 2]. Side effects, especially nephrotoxicity, of this drug limit its widespread use in high dose [3]. The need to develop new complexes with reduced nephrotoxicity and higher activity has stimulated the synthesis of many new complexes. Over the past years, a renewed interest in Pd(II) complexes as potential anticancer agents has developed. Though a number of interesting Pd(II) targets have been investigated [4–7], the biological utility of such agents continues to be questioned, this may be due to the poor solubility of common Pd(II) complexes under physiologic conditions. Many studies, including nucleic and amino acid derivatives, showed that 2-mercaptopyrimidine and 2-mercapto-4-aminopyrimidine are able to inhibit the synthesis of *t*-RNA [8]. Thus, they may act as valuable substrates in the synthesis of antitumor chemotherapeutic agents [9]. Also, the effect of 2-mercaptopyrimidine-5-carboxylic acid and S-analogy of pyrimidinic bases on oral epidermoid human carcinoma (KB) have been reported [10, 11].

As a continuation of our research in 4,6-diamino-5-hydroxy-2-mercaptopyrimidine complexes and their biological activities [12], in this research, we report the complexes obtained from the reaction of 4,6-diamino-5-hydroxy-2-mercaptopyrimidine (Hdahmp) with [Pd(bpy)Cl_2_] and [Ag(bpy)(H_2_O)_2_]NO_3_. They have been investigated using IR, ^1^HNMR, and mass spectra, conductivity and thermal measurements. In addition, the anticancer activity of these complexes against *Ehrlich ascites* tumor cells (EACs) has been reported.

## 2. EXPERIMENTAL

### 2.1. Material and methods

All manipulations were performed under aerobic conditions using 4,6-diamino-5-hydroxy-2-mercaptopyrimidine and all other reagents (Merck) as received. [Pd(bpy)Cl_2_] was synthesized by the literature method [13].

The cells of *Ehrlich ascites* (EACs) tumor were obtained from National Cancer Institute (Cairo, Egypt). After harvesting and preparation of the cells, their total number and viability were determined by counting using Trypan blue [14].

### 2.2. Instrumentation

Microanalyses were determined by the Micro Analytical Unit (Cairo University, Cairo, Egypt). Electronic spectra were recorded using a Unicam UV_2–100_ U.V.-vis. Spectrometer. IR spectra were measured as KBr discs on a Matson 5000 FT-IR spectrometer (Cairo University). ^1^H NMR spectra were measured on a Varian Gemini WM-200 spectrometer (Laser Centre, Cairo University). Thermal analysis measurements were made in the 20–800°C range at the heating rate of 10°C min^-1^, using *α*-Al_2_O_3_ as a reference, on a Shimadzu Thermogravimetric Analyzer TGA-50. Conductimetric measurements were carried out at room temperature on a YSI Model 32 conductivity bridge. Mass spectra were recorded on a Matson MS 5988 spectrometer (Micro Analytical Unit, Cairo University).

### 2.3. Synthesis of complexes

#### 2.3.1. [Pd(bpy)(dahmp)]Cl*·*H_2_O

To a stirred suspension of [Pd(bpy)Cl_2_] (0.17 g, 0.5 mmol) in methanol-benzene (3 : 2, V/V) (15 cm^3^), was added a methanolic solution of KOH (0.055 g, 1 mmol) containing Hdahmp (0.08 g, 0.5 mmol). The resulting suspension was stirred for two days and a brown complex was obtained. It was filtered off, washed with water and methanol, and then air-dried. Conductivity data (10^−3^ M in DMF): Λ_*M*_ = 97.0 ohm^−1^ cm^2^ mol^−1^. Elemental *Anal*. Calc. for C_14_ClH_15_N_6_O_2_SPd: C, 35.53; H, 3.17; N,17.76; S, 6.77; Cl, 7.51. Found C, 35.72; H, 3.11; N, 17.71; S, 6.85; Cl, 7.63.

#### 2.3.2. [Ag(bpy)(Hdahmp)]NO_3_

Silver nitrate (0.087 g, 0.5 mmol) in water (2 cm^3^) was added to bpy (0.078 g, 0.5 mmol) in methanol (35 cm^3^) to produce a colorless solution, to which Hdahmp (0.08 g, 0.5 mmol) was added. The reaction mixture was stirred in dark for 3 hours to produce a pale brown solid. It was filtered off, washed with little water, methanol, and diethyl ether, then dried in vacuo. Conductivity data (10^−3^ M in DMF): Λ_*M*_ = 60.0 ohm^−1^ cm^2^ mol^−1^. Elemental *Anal*. Calc. for C_14_H_14_N_7_O_4_SAg: C, 34.72; H, 2.89; N, 20.25; S, 6.61. Found C, 34.54; H, 2.78; N, 20.00; S, 6.42.

### 2.4. Conductivity measurements

The conductivity values for [Pd(bpy)(dahmp)]Cl and [Ag(bpy)(Hdahmp)]NO_3_ were determined. The compounds were dissolved in water and the measurements were done at concentrations; 1, 0.8, 0.6, 0.4, and 0.2 mM. The conductance values (Λ_*M*_) were calculated and plotted against concentration [15].

### 2.5. Anticancer activity against Ehrlich ascites carcinoma in mice

All the compounds were screened for their anticancer activity by dissolving samples in minimum amount of DMSO (Hdahmp) or water (complexes) and diluting with phosphate-buffered saline (PBS; pH = 7.2). The anticancer studies using *Ehrlich ascites* tumor cells (EACs) were carried out by incubating 0.2 mL of cells IP. All the treatments started 24 hours after inoculation for 45 days. The tumor-bearing mice were divided into three groups. Group (1) is the standard one that received the 5-florouracil [16] (5-fu; 20 mg/kg/day of mice) for comparison. Group (2) received Hdahmp, [Pd(bpy)(dahmp)]Cl, and [Ag(bpy)(Hdahmp)]NO_3_ complexes (0.01 mg/mice/day). Group (3) is the control one received physiological saline (0.9% sodium chloride).

## 3. RESULTS AND DISCUSSION

### 3.1. Synthesis of complexes

Table 1 lists two new complexes of 4,6-diamino-5-hydroxy-2-mercaptopyrimidine (Hdahmp). The elemental analyses of the isolated complexes agree with the assigned formula. The conductivities (Λ_*M*_) in DMF at room temperature showed the electrolytic character of these complexes [4, 16].

The complex [Pd(bpy)(dahmp)]Cl was prepared from [Pd(bpy)Cl_2_] and Hdahmp in methanol-benzene in presence of aqueous base, while [Ag(bpy)(Hdahmp)]^+^ was made from aqueous AgNO_3_ with bpy and Hdahmp in methanol.

The complexes are powder-like, stable in the normal laboratory atmosphere, and soluble in water, DMF, or DMSO. We had hoped to characterize the structure of one of the complexes by single X-ray crystallography, but were thwarted on numerous occasions by very small crystal dimensions. Thus, the characterization of these complexes was based on the physical and spectroscopic techniques.

### 3.2. Vibration spectra

**Scheme 1:**
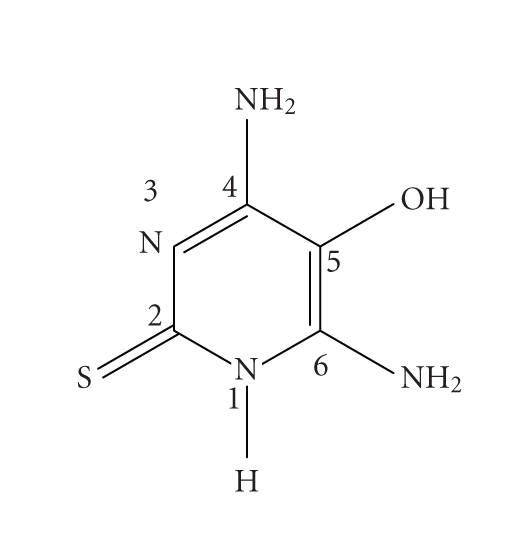
4,6-diamino-5-hydroxy-2-mercaptopyrimidine (Hdahmp).

The characteristic IR bands observed and vibration assignments of 4,6-diamino-5-hydroxy-2-mercaptopyrimidine (Hdahmp) complexes are reported in Table 1. The spectrum of Hdahmp supports the existence of the thione form in the solid phase (Scheme 1). This can be attributed to the presence of *ν*(NH) stretch at 2970 cm^−1^ [17], the absence of *ν*(SH) near 2600 cm^−1^, and the production of the characteristic thioamide bands due to extensive coupling of *δ*(NH), *ν*(C=N), *ν*(NCS), and *ν*(C=S) at 1652, 1562, 1455, and (1375, 1268, 1177) cm^−1^, respectively [18–20].

**Scheme 2:**
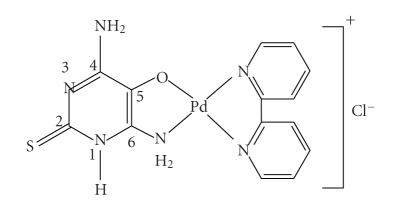
Structure of [Pd(bpy)(dahmp)]Cl.

**Scheme 3:**
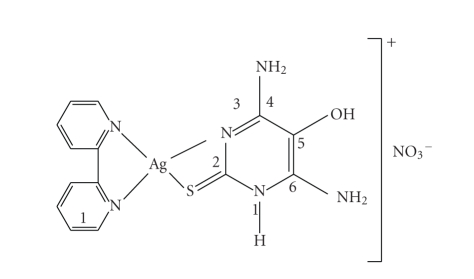
Structure of [Ag(bpy)(Hdahmp)_2_]NO_3_.

In the spectrum of [Pd(bpy)(dahmp)]Cl, the stretching vibration *ν*(OH) at 3305 cm^3^ in the free ligand is missing in the complex [21]. The bands at 3390 and 3185 cm^−1^, arising from *ν* ^*s*^(NH_2_) and *ν* ^*as*^(NH_2_), respectively [21], in the free ligand are shifted to lower wave numbers upon complexation [21, 22]. The bands arising from *ν*(C=N), *ν*(NCS), and *ν*(C=S) are not affected while the bands arising from *ν*(NH) and *δ*(NH) stretches are slightly shifted to lower wave number in the complexes. This suggests that Hdahmp acts as a mononegative bidentate ligand, coordinating the metal ion through the deprotonated hydroxyl and amino N(6)H_2_ groups, without any participation of the thione sulfur or cyclic nitrogen atoms in coordination [23]. This feature is further supported by the observation that a band near 1178 cm^−1^ arises from *ν*(C=S) stretch that remains unchanged [18] (Scheme 2). The vibrational spectrum of [Ag(bpy)(Hdahmp)]NO_3_ suggests the participation of the thione sulphur and the cyclic N(3) in coordination due to the shift observed in the *ν*(C=S) and *ν*(N–C=S) stretching vibrations (Scheme 3). This suggestion is supported by the slight shift of *ν*(NH) and *δ*(NH) to lower wave number with the existence of *ν* ^s^(NH_2_), *ν* ^as^(NH_2_), and *ν*(OH) stretches more or less in the same position as in the free ligand [18, 22, 24].

Also, the bands of the free bpy ligand near 740 cm^−1^ are shifted to higher frequencies in the complexes (773 cm^−1^) [4, 25].

The spectrum of [Ag(bpy)(Hdahmp)]NO_3_ shows new strong band near 1370 cm^−1^ assigned to the ionic uncoordinated NO_3_^−^ [16, 26, 27].

### 3.3. Electronic spectra

The electronic spectrum of [Pd(bpy)(dahmp)]^+^ complex shows bands at 475 and 326 nm due to ^1^A_1g_ → ^1^B_1g_ and ^1^A_1g_→^1^E_1g_, transitions in a square-planar configuration [4, 23, 27]. Also, the spectrum of [Ag(bpy)(Hdahmp)]^+^ shows bands at 467, 382, and 277 nm; the latter two may arise from charge transfer of the type ligand (*π*) → b_1g_ (Ag^+^) and ligand (*σ*) → b_1g_ (Ag^+^), respectively, in a typically distorted square planar environment around the metal ion [4, 28, 29].

### 3.4. ^1^H NMR spectra

The ^1^H NMR spectrum of Hdahmp in d_6_-DMSO shows two singlets at *δ*6.07 and 6.18 ppm arising from N(4)H_2_ and N(6)H_2_, respectively (Scheme 1). The proton of the hydroxyl group O(5)H appears as a broad singlet at *δ*9.13 ppm and the N(1)H proton gives a singlet at *δ*7.43 ppm. In the ^1^H NMR spectrum of [Pd(bpy)(dahmp)]^+^, the proton of the hydroxyl group is not observed while the resonance arising from N(6)H_2_ is shifted to lower field [24, 30]. Also, the resonances arising from N(4)H_2_ and N(1)H are slightly shifted to lower field. This is probably due to the decrease in the electron density caused by the withdrawing of electrons by the metal ions from the pyrimidine ring coordination centers [13, 23]. The ^1^H NMR spectrum of [Ag(bpy)(Hdahm)]^+^ confirms the neutral bidentate behavior of Hdahmp through the thione sulfur and the cyclic N(3) center, as there is a slight shift from the free ligand spectrum (Table 2). Also, the bpy ligand, in the complexes, shows upfield shifts as compared with [Pd(bpy)Cl_2_] or [Ag(bpy)(H_2_O)_2_]^+^. This is interpreted in terms of strong binding of dahmp^−^ to Pd(II) or Ag(I) in comparison to binding of chloride and aquo species, respectively [4–6].

### 3.5. Mass spectra

The mass spectrum of [Pd(bpy)(dahmp)]Cl*·*H_2_O shows a signal at *m/e* 474 (Cacld. 472.9) with 2.60% abundance. The spectrum shows signals at 355, 215, and 129 corresponding the loss of (H_2_O, C_2_H_3_N_3_S), (Cl, Pd) [4], and (N_2_, C_2_H_2_NO) fragments, respectively.

### 3.6. Thermal measurements

The thermal decomposition of [Pd(bpy)(dahmp)]Cl*·*H_2_O and [Ag(bpy)(Hdahmp)]NO_3_ was studied by using the thermogravimetry (TG) technique. The thermogram of [Pd(bpy)(dahmp)]Cl*·*H_2_O shows four TG inflections in the ranges 32–148, 150–360, 361–475, and 476–593°C. The first weight loss may arise from the elimination of crystal lattice water (Calcd. 3.81, Found 3.73%) [4]. The second step may arise from the release of half Cl_2_ and C_3_H_5_N_3_ fragments (Calcd. 25.06, Found 24.48%), the third step is due to the removal of CNS and half bpy (C_5_H_4_N) fragments (Calcd. 28.76, Found 29.22%) [27], while the fourth step is attributed to the removal of the other half of bpy species (Calcd. 16.49, Found 16.55%) followed by the formation of PdO at 665°C (Calcd. 25.88, Found 26.36%) [4, 27]. The thermogram of [Ag(bpy)(Hdahmp)]NO_3_ shows the first-step weight loss of 9.29% between 198 and 252°C, which corresponds to the release of NO_2_ species (Calcd. 9.51%). The second decomposition step occurs between 253 and 352°C, this weight loss is attributed the loss of C_3_H_6_N_3_ fragment (Calcd. 17.36, Found 17.89%). The third TG inflection between 432–520°C, may arise from the elimination of bpy, CS, three quarter species (Calcd. 49.18, Found 51.09 %), leaving Ag_2_O representing (Found 23.00%) [5, 27].

### 3.7. Conductivity measurements

**Figure 1:**
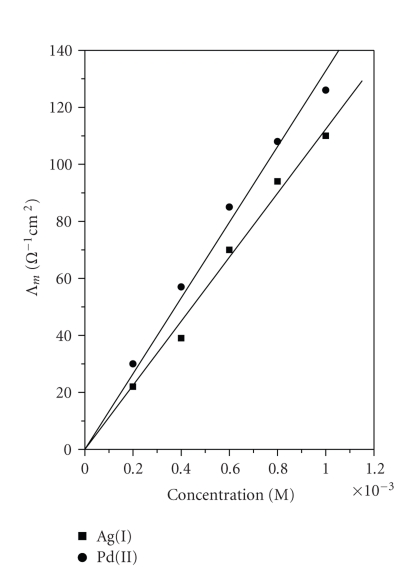
Conductance-concentration relationship of the complexes.

Figure 1 shows the plots of the conductivities of [Pd(bpy)(dahmp)]Cl and [Ag(bpy)(Hdahmp)]NO_3_ against concentrations. It is clear that as the concentration increases, the conductivity increases, indicating the complete ionization of the complexes species [31]. Since the conductivity for Cl^−^ and NO_3_^−^ is 76 and 71 ohm cm^2^, respectively [31, 32], this suggests that the complexes ionized completely in aqueous media [33].

### 3.8. Anticancerous activity

The reliable criteria for judging the efficacy of any anticancer drug are prolongation of life span, improving the clinical, hematological, and biochemical profile, as well as reduction in viable tumor cell count in the host [34, 35]. We have reported that [PdL(pa)]^+^, [PdL’(pa)Cl], and [Pd(bpy)(cdhp)] (*L* = 2,2′-bipyridyl; *L*′ = 9,10-phenanthroline, 2-(2′ -pyridyl)quinoxaline); pa = anion of 2-pepiridine carboxylic acid; cdhp = dianion of 5-chloro-2,3-dihydroxypyridine) in water or DMSO exhibit potent cytotoxic activity against *Ehrlich ascites* tumor cells [4, 5]. It is known that the anticancer available drugs inhibit the hematological and biochemical parameters (hemoglobin (Hb), red blood cells count (RBCs), and white blood cells count (WBCs); blood picture). The ultimate goal of this project is to develop mixed ligand complexes containing nitrogen bases effective against cancer without side effects on the hematological and biochemical parameters.

In order to detect the influence of Hdahmp, [Pd(bpy)(dahmp)]Cl, and [Ag(bpy)(Hdahmp)]NO_3_ on the hematological status of EAC-bearing mice, a comparison study was made among three groups of mice (each group contains seven mice) from the second day after inoculation. Group (1) tumor-bearing mice treated with 5-fu (standard [36, 37]). Group (2) tumor-bearing mice treated with Hdahmp, [Pd(bpy)(dahmp)]Cl, and [Ag(bpy)(Hdahmp)]NO_3_. Group (3) is the control tumor-bearing mice. The anticancer activity of Hdahmp, [Pd(bpy)(dahmp)]Cl, and [Ag(bpy)(Hdahmp)]NO_3_ shows remarkable efficacy manifested by survival and activity, as well as reduction in the tumor size. The hematological parameters including hemoglobin (Hb), red blood cells count (RBCs), and white blood cells count (WBCs) data are reported in Table 3. It is clear that the hematological parameters of tumor-bearing mice treated with Hdahmp, [Pd(bpy)(dahmp)]Cl, and [Ag(bpy)(Hdahmp)]NO_3_ exhibits much better significant figures with the use of small doses of (0.01 mg/mice/day) compared with the standard (5-fu), the market drug (*∼*0.4 mg/mice/day).

There are reports that complexes containing pyridine ring (cyclic nitrogen) display significant anticancer activity [4, 38]. Thus, the presence of the pyrimidine ring increases the anticancer activity and activates the binding of metal ion to the tumor DNA as it contains two cyclic nitrogen atoms [5].

The hematological parameters show that [Pd(bpy)(dahmp)]Cl and [Ag(bpy)(Hdahmp)]NO_3_ are more effective than Hdahmp itself, as the presence of both bpy and dahmp in the complexes possess a multiring planar area with nitrogen bases and hence higher hydrophobicity, which would lead the intercalation more deeply into the tumor DNA [5].

In order to investigate the action of Pd(II) and Ag(I) complexes in the tumor DNA, the intercalated complexes affecting the structure of the DNA prevent polymerase and other DNA binding proteins from functioning properly. As the complexes covantely bind to DNA with preferential binding to the N-7 position of guanine and adenine, they are able to bind two different sites on DNA, producing cross-links that cause increase in the viscosity in comparison to the normal unbound DNA. The results are prevention of DNA synthesis, inhibition of transcription, and induction of mutations [5, 39].

Regarding the tumor size and EAC count, in the control group was (220 × 10^6^ cells/cm^3^), reduced in using 5-fu to (80 × 10^6^ cells/cm^3^) while the strong reduction to 44.8 × 10^6^, 31.8 × 10^6^, and 30.4 × 10^6^ cells per cm^3^ was observed in using Hdahmp, [Pd(bpy)(dahmp)]^+^, and [Ag(bpy)(Hdahmp)]^+^, respectively. The strong reduction in EAC count and tumor size may be due to the reductive nature of many tumors that contain significant regions at low oxygen tension. Thus, they initiate a catalytic auto-oxidation process involving generation of reactive oxygen species. The coordinated dahmp and bpy may reduce toxic effects caused by xenobiotic core of the complexes, contribute to their anticancer action, and facilitate their transport through cell membrane [40]. Our investigations have shown that glutathione content, in liver and kidney, and glutathione-S-transferase activity were decreased, suggesting that the partial reduction products of oxygen, in the presence of the complexes, yield very reactive species, which could start catalytic oxidation of substrates and show antitumor action [41].

In order to detect the influence of the solvent in the cytotoxicity of Hdahmp, [Pd(bpy)(dahmp)]^+^, and [Ag(bpy)(Hdahmp)]^+^. As expected, the water-soluble [Pd(bpy)(dahmp)]^+^ and [Ag(bpy)(Hdahmp)]^+^ are less kidney toxic.

### 3.9. Effect of survival time

The mean survival time (MST) of groups 1 and 2 was compared with that of the control group using the following calculations [42]. Percentage (%) increase in lifespan over control = [(MST of treated group × 100/MST of control group) −100]; MST = days of each mice in the group/total number of mice. Percentage (%) increase in lifespan over control showed to be high in mice treated with Hdahmp, [Pd(bpy)(dahmp)]Cl, and [Ag(bpy)(Hdahmp)]NO_3_ (Table 3).

### 3.10. The side effects and toxicity

The side effects and toxicity of Hdahmp, [Pd(bpy)(dahmp)]^+^, and [Ag(bpy)(Hdahmp)]^+^ have been detected. After the first week of the treatment, the mice show flu-like attack and in the third week show spot dropping on the hair (alopecia). Fortunately, the solid organs have not been affected.

The study of detailed mechanism and in vivo anticancer screens (phase II & III) using the studied complexes are under way.

## 4. CONCLUSION

There are reports that complexes containing pyridine ring (cyclic nitrogen) display significant anticancer activity. The anticancer activity of the new water-soluble complexes, [Pd(bpy)(dahmp)]Cl and [Ag(bpy)(Hdahmp)]NO_3_, shows remarkable efficacy against *Ehrlich ascites* tumor cells (EACs) manifested by survival and activity, as well as reduction in the tumor size.

## Figures and Tables

**Table 1:** Spectral data of Hdahmp and its complexes.

			IR spectral data						
Complexes	*ν*(OH)	*ν* ^*s*^(NH_2_)	*ν* ^*as*^(NH_2_)	*δ*(NH)	*ν*(C=C) *ν*(C=N)	*ν*(NCS)	*ν*(CN) *ν*(NCS) *ν*(CS)	*ν*(M–O)	*ν*(M–N)
[Pd(bpy)(dahmp)]Cl	—	3397 3360	3165	1640	1556	1450	1362 1255 1176	524	409
[Ag(bpy)(Hdahmp)]NO_3_	3308	3396	3171	1650	1543	1479	1396 1277 1207	—	412 372*

*ν(Ag–S).

**Table 2:** ^1^H NMR spectral data of Hdahmp and its complexes.

Compounds	N(1)H	N(4)H_2_	O(5)H	N(6)H
Hdahmp	7.43	6.07	9.13	6.18
[Pd(bpy)(dahmp)]^+^	—*	6.08	—	6.46
[Ag(bpy)(Hdahmp)]^+^	—*	6.16	9.20	6.24

*Signal interefered with Ph or bpy protons.

**Table 3:** Haematological and biochemical parameters of Hdahmp and its complexes.

	Parameters					
Compound	Hb^(1)^ (12–16 g/dl)	RBCs^(2)^ (4.0–6.0 × 10^6^ cell/cm^3^)	HCT^(3)^ (35.0–50.0%)	WBCs^(4)^ (4000–11000 × 10^6^ cell/cm^3^)	EAC Count (× 10^6^ cell/cm^3^)	MSt/day^(5)^
Hdahmp	10.7	6.3	40.3	8800	44.8	13.2
[Pd(bpy)(dahmp)]Cl	11.4	6.4	40.3	12000	31.8	11.4
[Ag(bpy)(Hdahmp)]NO_3_	11.0	6.3	41	9000	30.4	11.2
5-fu	10.2	6.0	37.7	7600	80	13.6
Control (0.9% NaCl)	7.8	4.72	22.2	2400	220	9.0

^(1)^Hb = hemoglobin, ^(2)^RBCs = red blood cells count, ^(3)^HCT = hemato crate value, ^(4)^WBCs = white blood cells count values in normal mice are in parentheses, ^(5)^the mean survival time.
